# The APOE–Microglia Axis in Alzheimer’s Disease: Functional Divergence and Therapeutic Perspectives—A Narrative Review

**DOI:** 10.3390/brainsci15070675

**Published:** 2025-06-23

**Authors:** Aiwei Liu, Tingxu Wang, Liu Yang, Yu Zhou

**Affiliations:** 1 School of Basic Medical Sciences, Qingdao University, Qingdao 266071, China; 15866222086@qdu.edu.cn (A.L.); 3272683405@qdu.edu.cn (T.W.); 2 Department of Physiology, Binzhou Medical University, Yantai 264003, China; 3 Department of Physiology and Pathophysiology, School of Basic Medical Sciences, Qingdao University, Qingdao 266071, China; 4 Institute of Brain Sciences and Related Disorders, Qingdao University, Qingdao 266071, China; 5 School of Life Sciences and Health, University of Health and Rehabilitation Sciences, Qingdao 266000, China

**Keywords:** Alzheimer’s disease, apolipoprotein E, microglia, inflammation, phagocytosis, lipid metabolism

## Abstract

Apolipoprotein E (APOE) alleles play distinct roles in the pathogenesis of Alzheimer’s disease (AD), with *APOE*ε4 being the strongest genetic risk factor for late-onset AD, while *APOE*ε2 appears protective. Despite extensive research, the precise mechanisms by which *APOE* alleles contribute to AD pathology remain incompletely understood. Recent advances in multi-omics technologies and single-cell analyses have revealed that *APOE* alleles shape microglial phenotypes, thereby affecting amyloid clearance, inflammatory responses, tau pathology, and lipid metabolism. In this review, we provide a detailed overview of how *APOE* alleles differentially regulate microglial activation, inflammatory signaling, phagocytic activity, and lipid metabolism in the context of AD, with a particular focus on the *APOE*ε4-mediated disruption of microglial homeostasis via pathways such as TREM2 signaling, NF-κB/NLRP3 activation, ACSL1 upregulation, and HIF-1α induction. These insights not only advance our understanding of *APOE* allele-specific contributions to AD pathology, but also highlight novel therapeutic strategies targeting the APOE–microglia axis.

## 1. Introduction

Alzheimer’s disease (AD), the most prevalent neurodegenerative disorder and primary cause of dementia, is characterized by progressive memory loss, cognitive dysfunction, behavioral alterations, and mood disturbances. As the disease advances, patients gradually lose the ability to perform daily activities, ultimately becoming fully dependent on caregivers [1]. The prevalence of AD increases significantly with age, rising from approximately 10% in individuals over 65 to nearly 30% in those over 85. By 2050, it is projected that 152 million people will be affected by AD [2]. Beyond its profound impact on individuals, AD places a considerable strain on public health systems worldwide. As the global population ages, the morbidity and mortality rates associated with AD continue to rise, making it one of the top concerns for global healthcare. Despite extensive research, the exact cause of AD remains unclear [2,3]. Consequently, unraveling the pathogenesis of AD and developing novel diagnostic and treatment strategies are urgently needed. AD is primarily characterized by the extracellular accumulation of amyloid-β (Aβ) plaques, composed of Aβ peptides, along with the formation of neurofibrillary tangles (NFTs) resulting from hyperphosphorylated microtubule-associated protein tau [4]. Neuroinflammation also plays a significant role in AD pathology [5,6,7]. These pathological changes are closely associated with the severity of clinical symptoms and are influenced by various genetic factors [4,8].

Genetic factors play a crucial role in the pathogenesis of AD, with the *APOE* alleles being the most significant genetic risk factor for late-onset AD [9]. In peripheral tissues, APOE is mainly produced by the liver and macrophages. In the central nervous system (CNS), astrocytes serve as the primary cellular source of APOE under physiological conditions, where the protein is synthesized in the endoplasmic reticulum and Golgi apparatus before being secreted as lipoprotein particles [10]. Microglia, though not major producers under homeostasis, markedly upregulate APOE expression upon activation—particularly during aging or in response to AD pathology [11,12]. APOE participates in lipid and cholesterol transport within both the CNS and peripheral circulation through interactions with receptors (e.g., LDLR, LRP1, and HSPG) [13]. Beyond astrocytes and microglia, low-level APOE expression has been documented in other CNS cell types, including vascular pericytes, choroid plexus cells, and stress-activated neurons (e.g., following brain injury or during AD progression) [14].

In the human genome, the *APOE* gene comprises three main alleles—*APOE*ε2, *APOE*ε3, and *APOE*ε—which encode APOE2, APOE3, and APOE4, respectively. The differences between APOE isoforms lie in the amino acids at positions 112 and 158. Specifically, APOE2 has cysteine at both positions, APOE3 has cysteine at position 112 and arginine at position 158, and APOE4 has arginine at both positions [15]. There are significant differences in lipid affinity and binding efficiency with the LDLR family among different APOE subtypes. APOE4 has lower lipid affinity but higher binding affinity with LDLR family receptors; APOE3 shows moderate levels in both lipid affinity and receptor binding efficiency; APOE2 has the highest lipid affinity, yet the lowest binding efficiency with LDLR family receptors [16]. Genetic susceptibility to AD follows a clear hierarchy among *APOE* alleles, with *APOE*ε2 demonstrating protective effects, *APOE*ε3 representing neutral risk, and *APOE*ε4 conferring progressively stronger disease vulnerability. Approximately 40% of AD patients possess at least one *APOE*ε4 [17]. Carriers of one *APOE*ε4 copy exhibit a 3- to 4-fold increased risk of developing late-onset AD, typically with disease onset 2 to 5 years earlier. In contrast, individuals homozygous for *APOE*ε4 face a 9- to 15-fold higher risk and experience disease onset 5 to 10 years earlier than non-carriers [18,19]. Compared to *APOE*ε3/ε3 homozygotes, *APOE*ε2 carriers show an approximately 50% lower AD risk [17,20]. From an evolutionary perspective, *APOE*ε4 represents the ancestral allele conserved in primates and most mammals. During evolution, *APOE*ε3 first emerged, followed by *APOE*ε2. The later an isoform appears during evolution, the stronger its protective function seems to be [21]. The differential AD susceptibility associated with distinct *APOE* alleles reflects their isoform-specific modulation of AD pathogenesis. *APOE*ε4 drives AD progression through multifactorial mechanisms, including enhanced Aβ aggregation, exacerbated tau pathology, amplified neuroinflammation, and accelerated neuronal degeneration [22,23,24,25,26]. Conversely, *APOE*ε2 exerts neuroprotection by suppressing Aβ deposition, attenuating neuroinflammatory cascades, and resisting tau hyperphosphorylation [27,28,29,30,31]. While *APOE*ε3 typically maintains lipid homeostasis without significantly affecting AD progression [32], some rare variants (e.g., APOE3-Jac, APOE3ch) may paradoxically reduce AD risk by enhancing microglial clearance of tau pathology, suppressing neuroinflammation, and mitigating amyloid β toxicity [33,34].

Microglia play a complex role in AD pathology, exhibiting dual effects that depend on the pathological environment and their activation state. In the early stages of the disease, microglia become activated upon recognizing pathological substances such as Aβ plaques, releasing pro-inflammatory cytokines and chemokines to enhance the local immune responses. This activation facilitates microglial migration to sites of damage, where they phagocytose Aβ plaques, tau protein aggregates, and damaged neurons, thereby restricting the spread of pathological factors and preserving neural homeostasis [35,36,37,38,39]. However, as AD progresses, the continuous accumulation of Aβ and tau protein deposits persistently stimulates microglia by binding to multiple receptors, leading to their chronic activation. In this state, microglia release excessive inflammatory mediators and harmful substances [40]. This sustained inflammatory response not only exacerbates neuroinflammation but also accelerates Aβ deposition and promotes tau protein propagation, thereby driving further neurodegenerative changes [40,41,42,43]. Moreover, the accumulation of extensive NFTs pushes microglia into a detrimental inflammatory state, resulting in the phagocytosis of synapses and the secretion of neurotoxic factors. This harmful process ultimately causes neuronal damage and perpetuates a vicious cycle of neurodegeneration [35].

The APOE–microglia axis—a conceptual framework highlighting how APOE protein isoforms differentially modulate microglial phenotypes—has emerged as a key regulator of AD pathogenesis. As resident immune cells in the brain, microglia exhibit functions that are specifically regulated by APOE genotypes, which shape their functional attributes in neuroinflammation, phagocytosis, and metabolic homeostasis. Recent studies on this dynamic interplay have significantly advanced our understanding of AD, revealing how allele-specific microglial behaviors critically influence disease progression. Notably, translational research in this field relies on appropriate animal models. Wild-type mice possess only a single endogenous *Apoe* gene, fundamentally differing from the polymorphic *APOE* variants in humans. The advent of CRISPR/Cas9 gene editing has enabled the precise introduction of human *APOE*ε2/ε3/ε4 alleles into the murine genome, permitting mechanistic investigations into how these variants differentially modulate microglial function. Research has demonstrated that microglia carrying *APOE*ε4 (hereinafter referred to as *APOE*ε4 microglia) exhibit an exacerbated inflammatory response upon activation, resulting in aggravated neuronal damage [44,45]. Their phagocytic capacity is significantly impaired, most notably manifested through the deficient clearance of Aβ plaques [44,46,47,48]. Additionally, the migratory function of *APOE*ε4 microglia is impaired, causing delays in their arrival at injury sites in response to pathological changes [49,50]. These combined functional deficits synergistically contribute to the progression of AD [39]. In contrast, *APOE*ε2 microglia may demonstrate enhanced Aβ clearance and reduced release of pro-inflammatory factors, effectively mitigating neuroinflammation and promoting neuroprotection [27,46,51,52,53,54,55]. *APOE*ε3 maintains normal lipid metabolism, balanced inflammatory responses, and efficient Aβ phagocytic capacity in microglia [46,49,56] (Table 1). Based on these findings, this review aims to comprehensively evaluate the latest research on the influence of distinct *APOE* alleles on microglial function in AD and explore future research directions and potential clinical applications.

## 2. The Role of APOEε4 in Microglia Function Associated with AD

### 2.1. APOEε4 Regulates Microglial Activation States in AD Pathogenesis

Microglia, the primary immune cells of the CNS, play a pivotal role in neuroinflammation and neurodegenerative diseases [57]. Their activation state, which ranges from a ramified surveillance morphology to an amoeboid, reactive phenotype, is closely linked to disease progression [58]. In multiple microglial models, including those derived from AD transgenic mice, human-induced pluripotent stem cell (hiPSC), and mouse N9 microglia transfected with human *APOE* genes, *APOE*ε4 microglia exhibit reduced process branching, a shortened process length, and enlarged cell bodies, presenting an “activated” morphology [59,60,61]. In human brain tissue, researchers observed that the number and area occupied by activated microglia in the frontal and temporal cortices increased progressively with the number of *APOE*ε4 alleles [62]. Furthermore, using positron emission tomography (PET) and linear regression analysis, researchers demonstrated that the *APOE*ε4 genotype is significantly associated with microglial activation in medial temporal lobe regions (e.g., transentorhinal cortex and hippocampus) at early Braak stages (I-II), independent of Aβ plaques and tau pathology [63]. These results demonstrate that *APOE*ε4 promotes microglial activation. In addition, *APOE*ε4 affects the activation of microglia towards specific phenotypes in the AD pathological environment.

Traditionally, microglial activation has been classified into two categories: the pro-inflammatory M1 state and the anti-inflammatory M2 state [58]. However, in recent years, single-cell sequencing has identified *APOE* allele-associated microglial subpopulations in AD patients or mouse models (Table 2). In 5XFAD mice (a transgenic AD mouse model), researchers identified disease-associated microglia (DAM) [64], also known as neurodegenerative microglia (MGnD) [65], a distinct subpopulation induced by apoptotic neurons, β-amyloid plaques, or tau pathology [10,64]. DAM exhibit a distinct gene expression profile characterized by the upregulation of key markers such as *TREM2*, *APOE*, and *LPL*, alongside the downregulation of homeostatic genes like *CX3CR1*, *P2RY12*, and *TMEM119* [64]. These cells are predominantly localized to pathological regions, particularly around amyloid plaques, where they play dual roles in both neuroprotection and neurodegeneration [64,66,67]. On the one hand, DAM/MGnD microglia delay disease progression by clearing pathological aggregates (e.g., Aβ) through TREM2-dependent phagocytosis [65,68]. On the other hand, their chronic activation can lead to detrimental outcomes, such as excessive synaptic pruning mediated by the complement pathway [69,70] and the release of reactive oxygen species (ROS) [71] and pro-inflammatory cytokines like TNF-α, which exacerbate neuronal damage [72].

The effects of *APOE*ε4 on the DAM/MGnD phenotype exhibit significant context-dependence, with its regulatory direction varying across disease models and pathological stages. In the human microglial cell line HMC3, APOE4 (but not APOE2/3) specifically upregulates the expression of TREM2 and its downstream effector Clec7a, suggesting that *APOE*ε4 may enhance DAM/MGnD-related pathways under certain conditions [54]. Consistent with this, studies in APOE-targeted replacement (APOE-TR) mice demonstrate that aging alone can induce upregulation of DAM/MGnD genes and enrichment of DAM-like microglia against an *APOE*ε4 background, independent of AD pathology [77]. However, in APOE-knockout mice with microglia/central nervous system-associated macrophages (CAMs)-specific inducible expression of *APOE*ε4, *APOE*ε4 downregulates key disease-associated microglia (DAM)/microglial neurodegenerative phenotype (MGnD) genes (such as TREM2), thereby impairing the transition of microglia to the activated reactive state [46]. Similarly, data from APOE4 knock-in mice and AD patients further confirm that *APOE*ε4 negatively regulates the DAM/MGnD phenotype, suppressing the expression of critical DAM/MGnD genes, including Clec7a, Lgals3, and Spp1. Moreover, the selective removal of *APOE*ε4 in microglia rescues MGnD dysfunction in both APP/PS1 and MAPT P301S (PS19) transgenic mice [56], further supporting its inhibitory role. However, in MAPT P301S (PS19) transgenic mice, *APOE*ε4 drives pro-inflammatory gene expression, lysosomal dysfunction, and lipid metabolic abnormalities in microglia, inducing a neurotoxic phenotype that overlaps with DAM phenotype but has unique transcriptional characteristics [78,79].

**Figure 1:**
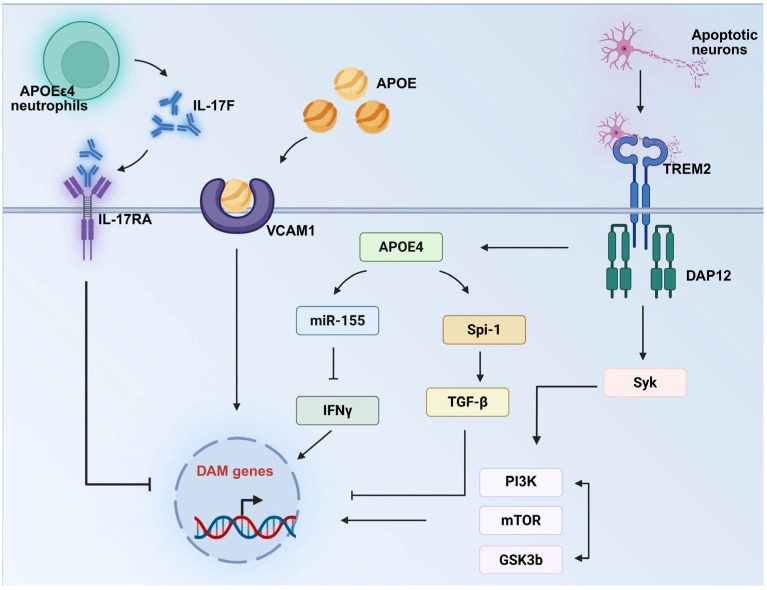
Molecular mechanisms by which APOE4 modulates the DAM/MGnD phenotype in microglia. In neurodegenerative conditions, phosphatidylserine exposed by apoptotic neurons activates the TREM2 receptor on microglia, initiating downstream signaling cascades through its interaction with DAP12. This triggers two key pathways: (1) SYK kinase phosphorylation directly upregulates DAM/MGnD-related genes and (2) collaboration with the APOE signaling pathway promotes DAM/MGnD phenotype acquisition. However, APOE4 disrupts this process via multiple mechanisms: (1) upregulating miR-155 to suppress IFNγ signaling, thereby impairing DAM activation; (2) increasing the expression of the transcription factor Spi-1 (PU.1), which activates the ITGB8-TGF-β pathway to antagonize the pro-DAM effects of TREM2-APOE. Notably, APOEε4 neutrophils exacerbate this suppression by releasing IL-17F, which engages microglial IL-17RA receptors. Separately, the APOE-VCAM1 interaction has been shown to promote DAM/MGnD phenotype formation.

The TREM2-APOE signaling axis plays a crucial role in regulating the DAM/MGnD phenotype of microglia in neurodegenerative disease models. TREM2, a transmembrane receptor expressed on microglia, can detect phosphatidylserine exposed by apoptotic cells or damaged neurons. In various mouse models of neurodegenerative diseases and human brain samples, researchers have discovered that TREM2 activation induced by apoptotic neurons triggers the APOE signaling pathway. The subsequent activation of this pathway mediates the transformation of microglia from a homeostatic phenotype to a neurodegeneration-associated DAM/MGnD phenotype [65]. TREM2 associates with the transmembrane adaptor DAP12(TYROBP); the TREM2-DAP12 receptor complex transmits intracellular signals through the protein tyrosine kinase SYK. This process is crucial for the activation of the DAM/MGnD phenotype [80]. Notably, the TREM2-APOE axis also activates miR-155 in DAM/MGnD microglia [65]. Paradoxically, as a pro-inflammatory microRNA, miR-155 suppresses the downstream IFNγ signaling pathway, thereby blocking the DAM/MGnD response [81]. This may be due to the TREM2-APOE axis preventing excessive inflammatory response during the early activation of MGnD phenotype. Moreover, through multi-dimensional studies on mouse genetic models (including gene knockout/knock-in) and human pathological samples, it has been confirmed that *APOE*ε4 inhibits the MGnD phenotype through the PU.1-ITGB8-TGFβ pathway, where the interaction between TGFβ and the TREM2-APOE axis serves as the core mechanism. Specifically, the ITGB8-TGFβ signaling pathway is vital for microglial homeostasis. *APOE*ε4 upregulates Spi-1 (PU.1), potentiates the ITGB8-TGFβ pathway, and suppresses the DAM/MGnD response. Meanwhile, TGFβ and the TREM2-APOE axis mutually inhibit each other to co-regulate DAM/MGnD microglial formation [56]. In addition, the IL-17F—IL-17RA axis also plays a role in the transition of microglia into the DAM/MGnD phenotype. In AD mouse models, the deletion of *APOE*ε4 in neutrophils (APOE4NTKO mice) reduced IL-17F expression in neutrophils, restored the microglial response to neurodegeneration, and limited plaque pathology. Blocking the 3IL-17F-IL-17RA axis with anti-IL-17F antibodies in AD mice promoted the transition of microglia to the DAM/MGnD phenotype, improved microglial phagocytic function, and alleviated cognitive impairment [82]. Furthermore, in IL-33-treated APP/PS1 mice, microglia upregulate the chemotactic protein VCAM1 (Vascular Cell Adhesion Molecule 1), promoting their migration toward Aβ plaques enriched with APOE. The interaction between VCAM1 and APOE drives microglial transition into a DAM/MGnD phenotype, characterized by enhanced phagocytic capacity and inflammatory signaling [83] (Figure 1). The investigation of these signaling pathways has uncovered a complex regulatory network underlying *APOE*ε4-mediated microglial dysfunction. Future research is needed to deeply elucidate the precise molecular mechanisms of each signaling pathway, particularly the cascade of upstream and downstream molecules within the TREM2-APOE axis and the regulatory details thereof. Additionally, a systematic analysis of the interactions between different pathways—including but not limited to the synergistic or antagonistic relationships between the TREM2-APOE axis and the IL-17F-IL-17RA axis, the ITGB8-TGFβ pathway, and others—is essential. By constructing integrated network models of these signaling pathways, we will gain a comprehensive understanding of the molecular basis of *APOE*ε4-induced microglial dysfunction, providing a theoretical foundation for the development of precision therapeutic strategies.

### 2.2. APOΕε4 Regulates Microglial Inflammatory Responses

*APOE*ε4 acts as a molecular amplifier of microglial reactivity. In the absence of inflammatory stimuli, compared to *APOE*ε2 and *APOE*ε3 microglia, *APOE*ε4 microglia exhibit elevated baseline secretion of tumor necrosis factor-α (TNF-α), thus indicating a primed pro-inflammatory state [84,85,86]. Following lipopolysaccharide (LPS) stimulation, *APOE*ε4 microglia further amplify their inflammatory response, secreting significantly higher levels of pro-inflammatory mediators, including TNF-α, interleukin-1β (IL-1β), and interleukin-6 (IL-6) [44,45,52,87]. Notably, this hyperreactivity is gene-dose-dependent, with homozygous *APOE*ε4 carriers showing more pronounced inflammatory responses than heterozygous individuals [52]. Interestingly, in APOE4-targeted replacement mice, basal pro-inflammatory gene expression (such as IL-1β, TNF-α, and NOS2) in female microglia is higher than that in male microglia, and the levels of secreted cytokines such as IL-1β, TNF-α, and IL-6 are significantly higher. This difference exists in the resting state without stimulation, and it further increases after inflammatory stimuli such as LPS and IFN-γ. Estrogen may amplify *APOE*ε4-induced pro-inflammatory gene transcription by enhancing the binding ability of NF-κB to DNA [85].

A study found that in the microglia of human brain tissues from *APOE*ε4 carriers, gene sets related to pro-inflammatory responses (such as T cell-mediated immune regulation, interferon-γ (IFN-γ) production, immune effector process regulation, etc.) were significantly upregulated, indicating that *APOE*ε4 microglia are in a state of continuously activated pro-inflammation [88]. In the normal aging human brain without neuritic plaques, researchers identified a set of “microglia-APOE cluster genes”, primarily associated with phagocytosis, pro-inflammatory responses, antigen presentation, opsonization, cytoskeletal regulation, and cellular motility. These genes exhibited coordinated upregulation in *APOE*ε4 carriers and downregulation in *APOE*ε2 carriers. Notably, in AD patients with neuritic plaques, the *APOE*ε4 carriers showed significant upregulation of these microglia-APOE cluster genes, yet no linear correlation with disease severity (Braak staging) was observed. These results suggest that *APOE*ε4 may prime microglia into a pro-inflammatory state through innate genetic factors rather than relying on pathological stimuli [89]. In patients with primary tauopathies (such as corticobasal degeneration [CBD], Pick’s disease, and progressive supranuclear palsy [PSP]) carrying *APOE*ε4, the pro-inflammatory gene sets in brain microglia—including those related to the TLR family, IL-1β, and IFN-γ signaling pathways—are significantly upregulated, with notably higher enrichment levels than those in *APOE*ε3 carriers [45].

However, a study has found that the inflammatory response of human *APOE*ε4 brains to AD pathology is significantly weakened, with the inflammatory gene expression profiles being highly similar to those of non-AD control (*APOE*ε3–control) brains. Moreover, microglia activation-related pathways (such as the SALL1-regulated quiescent-to-activated transition) and immune response pathways (such as cytokine signaling) are not significantly activated, suggesting an inability to clear Aβ plaques and tau tangles through inflammatory responses. In contrast, *APOE*ε3 brains exhibit a pronounced inflammatory response to AD pathology, in which microglia transition from a quiescent state to an activated state to combat AD pathology. This study is the first to reveal molecular evidence in human brain tissue that *APOE*ε4 leads to defects in AD-related inflammatory pathways [90]. Similarly, in both human brains (including those of AD patients) and AD mice (5 × FAD mice, a common mouse model of AD progression, crossed with mice carrying human *APOE*ε2, *APOE*ε3, or *APOE*ε4), researchers identified a special cell population called terminally inflammatory microglia (TIMs), which are characterized by ineffective inflammatory responses and impaired responses to chronic stimuli. The frequency of TIMs reaches 69% in 96-week-old AD*APOEε4 mice, while the frequency of TIMs in the brains of human AD patients increases with the progression of Braak stage and the carriage of *APOE*ε4, suggesting that their accumulation is driven by aging and *APOE*ε4 [73]. Based on these observations, we hypothesize that *APOE*ε4 drives a biphasic microglial response: early hyperactivation followed by late exhaustion. However, most animal models capture only the early hyperactivation phase, while human autopsy samples reflect the late exhaustion phase. This methodological gap obscures the dynamic regulatory role of *APOE*ε4 in AD pathogenesis. Collectively, these findings suggest that *APOE*ε4 creates a hyperresponsive but exhaustion-prone inflammatory system. The transition from hyperactivation to exhaustion raises important questions about the temporal dynamics of *APOE*ε4-mediated neuroinflammation. Are TIM a cause or consequence of disease progression? Furthermore, the reliance on post-mortem samples introduces potential artifacts, such as post-mortem interval effects, which may disproportionately impact *APOE*ε4 microglial transcriptomes. Future studies should prioritize longitudinal analyses, fresh-tissue validation, and advanced imaging techniques to unravel the spatiotemporal dynamics of *APOE*ε4 in AD pathogenesis.

*APOE*ε4 amplifies neuroinflammatory responses through two primary mechanisms: inflammatory pathway activation and metabolic reprogramming. These mechanisms collectively drive microglial hyperactivation and sustain chronic inflammation in AD.

#### 2.2.1. Inflammatory Pathway Activation

*APOE*ε4 enhances microglial inflammatory responses by hyperactivating key signaling pathways. Following LPS stimulation, *APOE*ε4 microglia exhibit stronger nuclear factor-κB (NF-κB) activation compared to *APOE*ε3 microglia [85]. APOE4 stimulation induces NF-κB activation and concurrently triggers NLRP3 inflammasome assembly, leading to the significant secretion of pro-inflammatory cytokines (TNF-α, IL-1β, and IL-6) from microglia. Additionally, APOE4 stimulation induces the generation of ROS through oxidative stress [91], which further amplifies NF-κB and NLRP3 inflammasome activation, creating a vicious cycle of inflammation [92,93]. Moreover, APOE4 stimulation impairs microglial mitophagy, leading to the accumulation of damaged mitochondria and the release of damage-associated molecular patterns (DAMPs). These DAMPs activate inflammasomes, further exacerbating NLRP3 neuroinflammation [91].

The TLR4-p38 MAPK pathway also plays a critical role. *APOE*ε4 microglia exhibit enhanced pro-inflammatory cytokine secretion through hyperactivation of the p38 MAPK pathway. Notably, treatment with the p38 MAPK-specific inhibitor SB203580 significantly suppressed cytokine production in *APOE*ε4 microglia compared to *APOE*ε2 microglia, suggesting genotype-dependent p38 mitogen-activated protein kinase (p38 MAPK) signaling sensitivity [52]. Similarly, the stimulation of microglia with LPS or oligomeric amyloid-beta 42 (oAβ42) activates the TLR4-p38 MAPK signaling pathway, triggering the release of pro-inflammatory cytokines. These effects are more pronounced in *APOE*ε4 microglia than in *APOE*ε3 microglia. Both TLR4 antagonists (e.g., LPS-RS and IAXO series) and p38α inhibitors (downstream effectors of TLR4) effectively suppress oAβ42-induced TNF-α secretion [94]. Notably, gender differences significantly influence TLR4-mediated neuroinflammation, with female *APOE*ε4 carriers exhibiting heightened sensitivity to inflammatory stimuli. The mechanism may be related to the higher TLR4 expression in females or more active downstream signaling (e.g., NF-κB) [95]. While these findings underscore *APOE*ε4-mediated regulation in driving neuroinflammation, most studies rely on LPS or Aβ stimulation models, which may not fully recapitulate the chronic, low-grade inflammation observed in human AD. Additionally, the gender-specific effects of *APOE*ε4 warrant further investigation. Future research is required to clarify how X-chromosome genes (such as TLR4 and CD14) or sex hormones (such as estrogen fluctuations) regulate the function of *APOE*ε4 in microglia; for example, through epigenetic or transcription factor regulation [85,95].

Moreover, a unique aspect of APOE4-mediated pro-inflammatory is its amino-terminal fragment, nAPOE4_1–151_, which binds to the TNF-α promoter, upregulating its expression and enhancing IL-1β secretion. This fragment also downregulates CXORF56, a homeostatic checkpoint gene, promoting microglial morphological activation and creating a positive feedback loop that sustains inflammation [96,97,98]. Paradoxically, full-length APOE4 may exert anti-inflammatory effects under certain conditions [99]. Notably, a seminal 2016 study employing multimodal experimental approaches (including molecular interaction assays, genomic analyses, and animal models) provided the first definitive evidence that APOE, particularly the APOE4 isoform, can function as a transcription factor by directly binding DNA to modulate gene expression [100]. Although nAPOE4_1–151_ exhibits characteristics resembling those of a transcriptional regulator, these experiments do not confirm its identity as a bona fide transcription factor [96,97,98,99]. These findings elucidate previously underappreciated, non-canonical functions of APOE that extend beyond its well-characterized role in lipid metabolism. Furthermore, recent studies have revealed that LilrB3, an immune checkpoint receptor protein expressed on the surface of microglia, serves as a specific receptor for APOE4, exhibiting minimal binding affinity for APOE2. The specific interaction between APOE4 and LilrB3 activates microglia, driving their transition into a pro-inflammatory state [101]. Additionally, LPS activates cholesterol 25-Hydroxylase (CH25H) expression in microglia through the TLR4 pathway, catalyzing the conversion of cholesterol to 25-hydroxycholesterol (25-HC) and significantly increasing its production. During this process, the *APOE*ε4 genotype further enhances the induction efficiency of CH25H, causing *APOE*ε4 microglia to produce significantly higher levels of 25-HC than *APOE*ε2/3 microglia after LPS stimulation. The generated 25-HC promotes IL-1β maturation and secretion by activating the NLRP3/caspase-1 inflammasome. *APOE*ε4 microglia are more sensitive to this process. The above mechanisms form a positive feedback loop in the *APOE*ε4 background: *APOE*ε4 not only promotes 25-HC production by enhancing CH25H expression but also improves the sensitivity of microglia to 25-HC-mediated inflammatory responses through unknown mechanisms, ultimately leading to exponentially exacerbated IL-1β-mediated neuroinflammation [102].

#### 2.2.2. Metabolic Reprogramming

**Figure 2:**
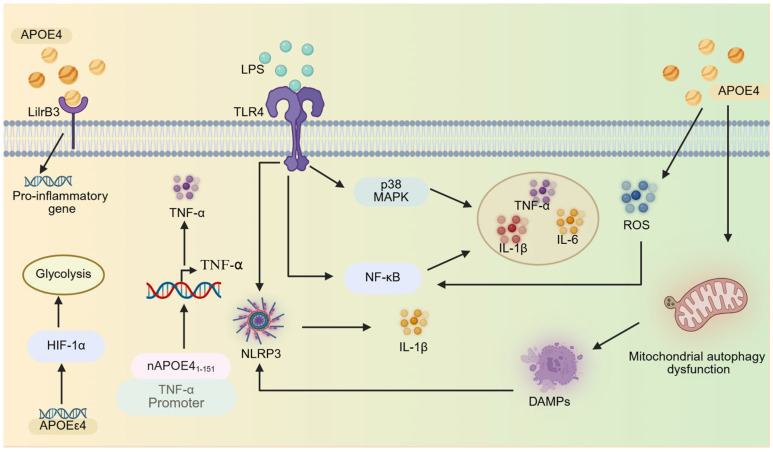
Inflammatory signaling pathways involved in microglial regulation by *APOE*ε4. In microglia, LPS binding to TLR4 activates NF-κB and p38 MAPK signaling pathways, driving pro-inflammatory cytokine release (TNF-α, IL-1β, IL-6) and NLRP3 inflammasome-dependent IL-1β secretion—processes significantly exacerbated by *APOE*ε4. Additionally, APOE4 disrupts mitochondrial autophagy, leading to ROS accumulation, which amplifies inflammatory signaling through NF-κB positive feedback, while mitochondrial damage releases DAMPs that further activate inflammasomes. Furthermore, the APOE4 proteolytic fragment nAPOE41–151 directly binds the TNF-α-promoter to enhance its expression, and APOE4-LilrB3 interaction promotes microglial activation and pro-inflammatory polarization. Notably, *APOE*ε4 also enhances HIF-1α activity, inducing glycolytic reprogramming that sustains microglial inflammatory responses.

The second major mechanism involves the *APOE*ε4-mediated regulation of microglial energy metabolism, which creates an energy landscape favoring sustained cytokine production [77]. Specifically, under steady-state conditions, microglia primarily rely on oxidative phosphorylation for energy generation [103,104]. However, compared to *APOE*ε3, *APOE*ε4 triggers a metabolic shift toward glycolysis during inflammation. This shift is marked by upregulated glycolysis-related genes and suppressed oxidative phosphorylation pathways. This metabolic shift provides an energetic foundation for sustained cytokine production, as glycolysis enables rapid ATP generation to meet the heightened energy demands of cells during inflammatory responses [77]. Mechanistically, this shift is driven by the enhanced activity of hypoxia-inducible factor 1α (HIF-1α), which promotes glycolytic flux and suppresses mitochondrial respiration [45,77,105] (Figure 2). However, the causal relationship between glycolytic predominance and microglial hyperactivation remains unresolved, with current evidence being insufficient to determine whether this metabolic reprogramming initiates or results from excessive immune activation [105].

### 2.3. APOEε4 Regulates Microglial Phagocytosis in AD

In various AD models, *APOE*ε4 microglia demonstrate significant impairments in phagocytosing Aβ aggregates, leading to their pathological accumulation in the brain [46,48,60]. Notably, in human *APOE*ε4 neuron-engrafted chimeric mice, microglial depletion resulted in an approximately 50% reduction in the average number of Aβ aggregates, demonstrating a synergistic interaction between *APOE*ε4 and microglia in promoting Aβ aggregate formation [47].

In both AD mouse models and human brains, microglia associated with Aβ plaques typically exhibit a DAM/MGnD phenotype, characterized by the upregulated expression of phagocytosis-related genes and the presence of engulfed Aβ particles [64,65]. Research demonstrates that the TREM2-APOE signaling axis plays a pivotal regulatory role in this process—Aβ binding to TREM2 receptors triggers downstream signaling cascades that promote microglial transition to the DAM/MGnD phenotype, thereby enhancing Aβ phagocytic clearance and effectively mitigating its neurotoxic effects [46,64,65,106]. However, this protective mechanism is significantly impaired in the *APOE*ε4 context. Under Aβ plaque conditions, *APOE*ε4 microglia show a markedly compromised transition to the DAM/MGnD phenotype, likely due to the disrupted TREM2-APOE signaling axis [46,56]. Although TREM2 and its signaling adaptor TYROBP expression positively correlates with *APOE*ε4 in microglial-like cells [60], *APOE*ε4 downregulates TREM2 expression in E4FAD mice. This impairment compromises microglial recognition and the binding of Aβ deposits, consequently disrupting protective barrier formation and plaque compaction [107]. TREM2 deficiency further exacerbates microglial barrier dysfunction, reducing both plaque-associated microglial numbers and their Aβ-encapsulating capacity [108,109]. Notably, the impact of TREM2 deficiency exhibits allele-specific effects, with *APOE*ε4 microglia demonstrating more pronounced phagocytic deficits compared to *APOE*ε3 microglia [49,108]. This genotype-dependent phenomenon underscores the critical importance of considering genetic background when developing TREM2-targeted therapeutic strategies.

Additionally, researchers have identified two distinct microglial subpopulations associated with amyloid plaques: ARM and MHC-II microglia. Amyloid-responsive microglia (ARM) are a specific subpopulation identified in post-mortem human brain tissue, particularly in the middle frontal neocortex. ARM counteract Aβ deposition through protective barrier formation and enhanced phagocytic capacity, characterized by high expression of Cluster of Differentiation 163 (CD163), a scavenger receptor that facilitates the receptor-mediated endocytosis of hemoglobin–haptoglobin complexes following hemorrhage [74]. In the brain tissue of Alzheimer’s patients, CD163-positive microglia are often found in close proximity to Aβ plaques, especially neuritic plaques, and exhibit increased expression of CD68, a lysosomal marker [110]. ARM are more abundant in AD patients carrying the *APOE*ε3/ε3 genotype, while their numbers are significantly reduced in *APOE*ε4 carriers and individuals with the TREM2-R47H variant [74]. Using an in vivo chimeric AD model (hiPSC-derived neurons transplanted into the hippocampus of APOE knock-in mice), researchers identified another microglial subpopulation, MHC-II microglia, which is characterized by high expression of MHC-II molecules and pro-inflammatory genes. Notably, neuronal *APOE*ε4 drives the enrichment of microglial subsets with elevated MHC-II expression. Strikingly, even in mice with an *APOE*ε4 genetic background, the proportion of MHC-II+ microglial subsets is significantly decreased when neurons lack *APOE*ε4 (hEKO-E4KI group) [47]. Previous studies indicate that MHC II-positive microglia accumulate around amyloid plaques and NFTs in both AD mouse models and human AD brains [111,112]. Researchers found that the intracerebroventricular (ICV) injection of amyloid β (Aβ)-specific T helper 1 (Aβ-Th1) cells into 5XFAD mice induces the differentiation of MHCII+ microglia. MHCII+ microglia exhibit a stronger capacity to clear Aβ plaques compared with MHCII− microglia [113]. In a recent study, hiPSC-derived neurons carrying *APOE*ε4 (hE4) were transplanted into the hippocampus of APOE4 knock-in (E4KI) mice to establish a human–mouse chimeric model. The results revealed a novel mechanism by which *APOE*ε4 promotes the progression of Aβ deposition and tau pathology through remodeling microglial phenotypes (such as inducing high-expression MHC-II subsets) and forming “toxic interactions” with neurons [114].

*APOE*ε4 significantly influences microglial activation, driving their differentiation into specific phenotypes, such as DAM/MGnD, ARM, and MHC-II microglia. However, the diminished phagocytic capacity of *APOE*ε4 microglia is not solely attributable to reduced activation potential. There exist additional mechanisms underlying *APOE*ε4-mediated deficits in phagocytic function. In E4FAD mice, although reactive microglia accumulate around Aβ plaques, they display a dystrophic morphology characterized by swollen somata and thickened processes, significantly compromising phagocytic function [115]. In addition, in vivo and in vitro, *APOE*ε4 microglia exhibit significant lysosomal impairment, characterized by aberrant lysosomal trafficking and impaired endosome-lysosome activity [78,84,116]. Under normal conditions, microglia internalize Aβ via endocytosis, followed by the fusion of endosomes with lysosomes, where Aβ is efficiently degraded by hydrolases [117,118]. However, lysosomal impairment severely compromises this clearance process, leading to the accumulation of Aβ within lysosomes [55]. The Aβ accumulated in lysosomes exhibits a “seeding effect,” which can induce further Aβ aggregation in vitro, suggesting that lysosomal dysfunction may trigger Aβ aggregation and deposition [119]. Despite these insights, the precise molecular mechanisms linking *APOE*ε4 to lysosomal impairment remain incompletely understood. Notably, both APOE3 and APOE4 facilitate Aβ binding to the cell surface and promote its lysosomal trafficking and degradation, with APOE3 exhibiting a more pronounced effect [119]. In the co-culture model of brain sections from 5XFAD transgenic mice and N9 microglia stably transfected with human *APOE*ε4, as well as in the in vitro functional validation model of *APOE*ε4/N9 microglia, a recent study found that *APOE*ε4 impairs the clearance of Aβ by microglia by inhibiting autophagic flux and mitochondrial function. Autophagy inducers such as rapamycin can reverse this defect by enhancing autophagy and mitophagy. This study reveals a novel mechanism by which *APOE*ε4 promotes pathological progression in AD through the autophagy–mitochondria axis [114].

**Figure 3:**
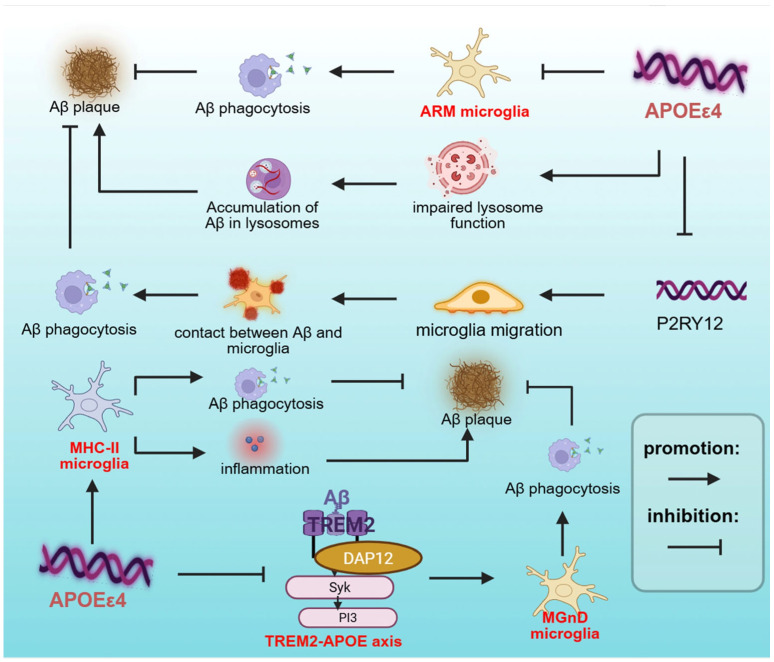
*APOE*ε4 disrupts microglial Aβ clearance through multiple convergent mechanisms. APOEε4 promotes the formation of MHC-II microglia, which exhibit enhanced inflammatory responses and Aβ phagocytosis—the former promotes, while the latter suppresses, Aβ plaque accumulation. Additionally, *APOE*ε4 inhibits the TREM2-APOE axis, impairing the formation of MGnD and ARM, both of which enhance Aβ clearance. *APOE*ε4 also disrupts microglial lysosomal function, leading to intracellular Aβ accumulation and promoting plaque formation. Furthermore, APOEε4 downregulates P2RY12, impairing microglial chemotaxis and the ability of microglia to form protective barriers around plaques.

Furthermore, *APOE*ε4 compromises microglial chemotaxis toward Aβ plaque due to the decreased expression of the P2RY12, which is crucial for microglial protrusion extension and plaque coverage. As an ATP receptor, P2RY12 is crucial for microglial protrusion migration in response to Aβ [49,50] (Figure 3). Notably, isoform-specific lipidation states critically regulate Aβ clearance efficiency. Lipidated APOE2 enhances Aβ clearance, whereas the reduced lipidation efficiency of APOE4 impairs this process, conferring a functional disadvantage [55]. In addition, *APOE*ε4 also has a cross-cellular regulatory effect. In the APP/PS1 mouse model, *APOE*ε4 expression attenuates LGALS3-dependent microglia–astrocyte crosstalk, leading to defective astrocytic plaque encapsulation and clearance. Strikingly, microglia-specific *APOE*ε4 knockout rescues LGALS3 signaling, reactivating astrocytic phagocytic function and improving plaque clearance [56].

*APOE*ε4 not only limits the ability of microglia to engulf and clear Aβ, but also regulates their response to tau protein. In chimeric mice transplanted with human *APOE*ε4 neurons, the impact of microglia on tau pathology is dependent on *APOE* genotype. In the presence of *APOE*ε4, microglia play a significant role in promoting tau pathology, as evidenced by the significantly reduced p-tau deposition in the hippocampus following microglial depletion in *APOE*ε4 chimeric mice. By contrast, *APOE*ε3 does not exhibit this effect [47]. Additionally, *APOE*ε4 microglia exhibit pronounced lysosomal abnormalities, including increased lysosomal mass and abnormally reduced baseline pH (hyperacidification). Notably, upon myelin stimulation, these cells fail to exhibit the expected further acidification, instead showing minimal pH changes or even slight alkalinization [84]. Given that lysosomal hydrolytic enzymes require an acidic environment (pH 4.5–5.0) for optimal activity, this dysregulated acidification directly compromises microglial tau degradation capacity, leading to intracellular tau accumulation [120]. If lysosomes fail to degrade the engulfed tau, the undigested tau is then released into exosomes, which are secreted by microglia. These exosomes transfer pathological tau to neurons, where the exogenous tau interacts with endogenous tau, promoting its aggregation and misfolding [121,122]. Additionally, in the *APOE*ε4 context, TREM2 deficiency exacerbates tau-mediated neurodegeneration and tau pathology. While *TREM2* knockout attenuates certain TREM2-dependent reactive gene expression in microglia, it preserves the elevated expression of TREM2-independent genes (e.g., lysosomal functional markers *CTSB* and *CTSD*). These findings demonstrate that, under *APOE*ε4 conditions, tau pathology drives a TREM2-independent microglial proliferative response that actively promotes tau-mediated neurodegeneration [123].

*APOE*ε4 also affects the ability of microglia to phagocytose and degrade other substrates, thereby influencing the progression of AD. In APOE-TR mice, during cuprizone (CPZ)-induced demyelination, microglia in *APOE*ε4 mice exhibited reduced efficiency in clearing the myelin debris that are abundant in lipids compared to *APOE*ε2 mice [53]. Although AD is not typically considered a demyelinating disorder, focal demyelination has been observed in both AD patients and mouse models, and it is associated with Aβ and neurofibrillary pathology [124,125]. Additionally, the phagocytic ability of microglia in APOE4 knock-in mice against apoptotic neurons is significantly lower than that of microglia in APOE3 knock-in mice, and they fail to upregulate the expression of genes related to engulfment when encountering apoptotic neurons [56]. Moreover, microglia in APOE4 knock-in mice exhibit significantly enhanced phagocytosis of synaptic materials compared to those in APOE3-KI mice. Mechanistically, APOE4 induces upregulation of MHC-I molecules on the surface of neurons. This abnormal expression is recognized by microglia as an “eat-me” signal, thereby inducing excessive synaptic phagocytosis by microglia [126]. Notably, compared with APOE4 knock-in mice, microglia in the hippocampal tissue of the P301S/APOE4 knock-in mouse model exhibit significantly enhanced synaptic phagocytosis, and their excessive synaptic phagocytosis is an important factor leading to synaptic loss in AD [78]. Furthermore, astrocyte-derived APOE4 may promote microglial synaptic clearance in tauopathy. In the tau pathology mice model, the study found that the deletion of astrocytic *APOE*ε4 reduced the expression of disease-associated microglia (DAM/MGnD) genes (such as Clec7a, H2-D1, and Spp1), increased the expression of homeostatic genes (such as P2ry12), and decreased the phagocytosis of synaptic elements by microglia [10].

### 2.4. APOEε4 Regulates Microglial Lipid Metabolism

APOE plays a central role in lipid metabolism within microglia, primarily through its ability to bind lipids and form lipoprotein particles, which facilitate intercellular lipid transport [127]. APOE regulates cholesterol metabolism in microglia through interacting with receptors such as the low-density lipoprotein receptor (LDLR), low-density lipoprotein receptor-related protein 1 (LRP1), and TREM2. These interactions mediate cholesterol uptake and clearance, maintaining intracellular lipid homeostasis [128]. Extensive research has demonstrated that *APOE*ε4 disrupts microglial lipid homeostasis and exacerbates the accumulation of lipid droplets (LDs) in microglia. During aging and AD, microglia accumulate LDs, which are primarily composed of cholesterol, triglycerides, and their esters [76,105,129]. However, *APOE*ε4 microglia exhibit significantly greater LDs accumulation compared to *APOE*ε3 microglia [46,56,76,126]. This phenomenon is observed even in the absence of aging or AD pathology, suggesting an intrinsic lipid dysregulation in *APOE*ε4 microglia [46,84,105,129]. Notably, transient receptor potential vanilloid 1 (TRPV1) is a ligand-gated ion channel capable of reversing APOE4-induced LDs accumulation in microglia [126].

The mechanisms underlying *APOE*ε4-mediated LDs accumulation are multifaceted. In hiPSC-derived *APOE*ε4 microglia, genes associated with cholesterol biosynthesis (including *HMGCR*, *SREBF2*, and *SCAP*) were significantly upregulated, while the expression of cholesterol efflux-related genes (such as ABCA1 and ABCG1) was downregulated. Additionally, reduced expression of lysosomal-related genes (e.g., *LIPA*) indicates impaired lysosomal function and compromised cholesterol and lipid degradation [130]. Furthermore, in induced microglia-like cells (iMGLs) expressing *APOE*ε4, genes involved in lipid synthesis (e.g., *ACSL1*) are upregulated, whereas those related to fatty acid β-oxidation (e.g., *ACOX1*, *CPT1A)* and lipid catabolism (e.g., *LIPA*, *SMPD1*) are downregulated, accompanied by reduced lysosomal acid lipase activity. This results in the inefficient degradation of LDs, exacerbating lipid accumulation [105]. In *APOE*ε4 microglia, the accumulation of LDs is associated with increased lysosomal mass and acidic pH, which may impair lysosomal lipid degradation efficiency, thereby forming a vicious cycle of lipid accumulation—lysosomal dysfunction—further lipid accumulation [84]. Moreover, APOE exhibits isoform-dependent cholesterol efflux capacity, with the following order: APOE2 > APOE3 > APOE4. APOE mediates cholesterol efflux through ATP-binding cassette transporter A1 (ABCA1), facilitating the formation of high-density lipoprotein (HDL) particles and consequently reducing intracellular cholesterol levels in microglia. Due to its having the lowest cholesterol efflux efficiency, APOE4 results in the most pronounced intracellular cholesterol accumulation in microglia [131]. Additionally, APOE4 and tau pathology synergistically disrupt cholesterol metabolism in microglia through dual mechanisms involving enhanced synthesis and impaired lysosomal degradation. This interaction upregulates cholesterol biosynthesis genes (e.g., *HMGCR*) via SREBP2 activation, thereby promoting endogenous cholesterol production. Simultaneously, it suppresses the LXR pathway, disrupting its negative feedback regulation of SREBP2. Furthermore, the combined effects of APOE4 and tau significantly reduce lysosomal acid lipase (LIPA) activity, impairing the degradation of both cholesteryl esters and phosphatidylcholines [78].

In aged mice, GRN knockout mice (a model for frontotemporal dementia, FTD), and brain tissues from normal human aging, researchers have identified a special subpopulation of microglia: lipid-droplet-accumulating microglia (LDAM). These cells exhibit massive intracellular accumulation of LDs. Moreover, these cells also show severe functional defects, such as decreased phagocytic ability, elevated ROS production, and a pro-inflammatory phenotype [75]. Subsequently, researchers identified LDAM in the brain tissues of AD patients, particularly in those with the *APOE*4/4 genotype, and proposed that the formation of LDAM is closely associated with acyl-CoA synthetase long-chain family member 1 (ACSL1) expression in microglia. ACSL1 is a key enzyme in lipid droplet biogenesis, and the overexpression of ACSL1 is sufficient to induce the formation of triglyceride-specific lipid droplets in multiple cell types. In iPSC-derived microglia, fibrillar Aβ (fAβ) induces ACSL1 expression, triglyceride synthesis, and LDs accumulation, with this induction being more pronounced against the APOE4 background. Notably, LDAM exhibits defective phagocytic function, lysosomal accumulation, and secretion of inflammation-related chemokines [76].

Growing evidence suggests that the *APOE*ε4-mediated dysregulation of microglial lipid metabolism is linked to its inflammatory response and impaired phagocytic function. Accumulating evidence demonstrates an interplay between Aβ and microglial lipid metabolism, which is markedly enhanced in the *APOE*ε4 context. In the presence of Aβ, microglial LDs accumulation is exacerbated, particularly in *APOE*ε4 microglia [46,56]. In the brains of AD patients, especially those carrying the *APOE*ε4/ε4 genotype, lipid bodies resembling LDs have been observed. These lipid bodies are typically located near Aβ plaques and may exist within or around microglia. The number of lipid bodies is positively correlated with the number of Aβ plaques, the level of tau pathology, and the expression of the *ACSL1* gene [76]. In addition, in vivo and in vitro models, Aβ stimulation leads to the accumulation of LDs in microglia, accompanied by an upregulation of ACSL1 expression, which is more pronounced against the *APOE*ε4/ε4 background. Mechanistically, in *APOE*ε4 microglia, NF-κB hyperactivation enhances *ACSL1* transcription via direct binding to its promoter [46,76]. Based on the above findings, we conclude that Aβ pathology promotes the accumulation of LDs in microglia, particularly in *APOE*ε4 microglia, and the ACSL1 may be a potential mediator of this process. Intriguingly, dysregulated microglial lipid metabolism, particularly cholesterol metabolism, can in turn influence Aβ pathology. Microglial intracellular cholesterol levels regulate Aβ trafficking to late endosomes/lysosomes for degradation. Reduced intracellular cholesterol enhances late endosomal/lysosomal transport efficiency by promoting Rab7 recycling via guanine nucleotide dissociation inhibitor (GDI)-mediated mechanisms. Specifically, cholesterol depletion facilitates Rab7 membrane dissociation and subsequent recycling, thereby accelerating the transport of Aβ-containing vesicles to lysosomes [131]. As previously discussed, *APOE*ε4 microglia exhibit more pronounced LDs accumulation and elevated intracellular cholesterol levels, which consequently impair Aβ lysosomal trafficking and degradation. Recent studies have revealed a novel cholesterol-induced mechanism of Aβ pathology. Lipidated APOE (cholesterol-bound form) is efficiently endocytosed by microglia via the LDLR and subsequently enters the endolysosomal system. Within the acidic environment of lysosomes, APOE undergoes delipidation and conformational changes, forming β-sheet-rich fibrillar aggregates (amyloid-like fibrils). This process is directly linked to lysosomal dysfunction, as delipidated APOE cannot be effectively cleared. Intracellular cholesterol depletion exacerbates lysosomal impairment, significantly promoting APOE aggregation. These APOE aggregates directly induce Aβ fibrillization and deposition within microglia. Furthermore, through microglial cell death or extracellular vesicle release, APOE aggregates can be transmitted to neighboring cells, thereby propagating Aβ pathology [132].

**Figure 4:**
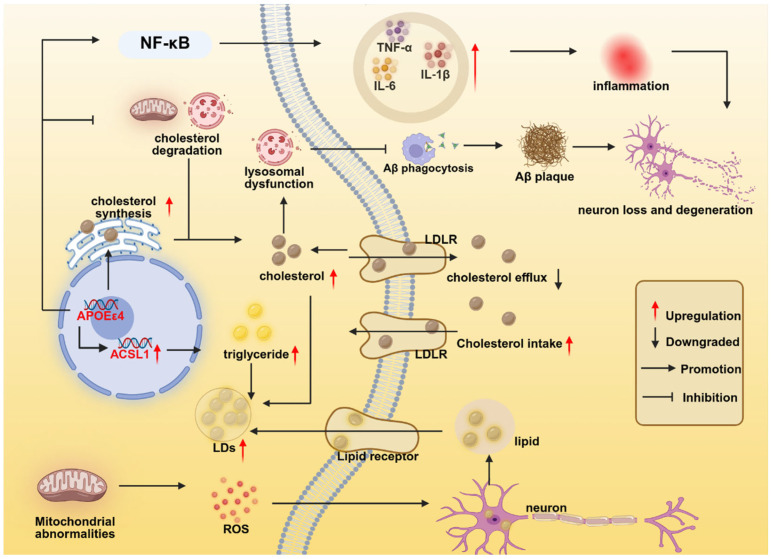
*APOE*ε4 disrupts microglial lipid metabolism, linking it to inflammation and phagocytic deficits. *APOE*ε4 disrupts microglial lipid metabolism by increasing ACSL1-mediated triglyceride synthesis while simultaneously promoting cholesterol uptake and biosynthesis but suppressing cholesterol degradation and efflux, ultimately leading to intracellular cholesterol accumulation and lipid droplets (LDs) formation. The resulting lipid-laden microglia exhibit elevated ROS production, which induces neuronal lipid release, thereby further exacerbating microglial LDs accumulation, establishing a vicious cycle. Moreover, LDs accumulation promotes NF-κB pathway activation with consequent pro-inflammatory cytokine release, whereas cholesterol accumulation impairs lysosomal function and compromises Aβ clearance. These pathological changes collectively drive sustained neuroinflammation, Aβ plaque deposition, and, ultimately, neuronal degeneration and death.

LDs accumulation promotes microglia to exhibit enhanced neuroinflammatory effects, and this effect is amplified in the *APOE*ε4 background. Studies have found that LDAM rich in LDs produce high levels of ROS and release pro-inflammatory cytokines [75]. In human-induced pluripotent stem cell-derived microglia (iMG) with *APOE*ε4/4 and *APOE*ε3/3 genotypes, *APOE*ε4/4 iMG enriched in LDs exhibit more pronounced inflammatory responses. Transcriptome analysis shows that compared with LDs–low iMG, LDs–high iMG of *APOE*ε4/4 have higher expression of NF-κB-related pro-inflammatory cytokines (such as TNFA and IL1B), and the NF-κB pathway is significantly activated [76]. In APOE-TR mice and microglial cell line BV2, LDs accumulation activates microglia through the NF-κB pathway, inducing the expression of MHC-II and the secretion of pro-inflammatory factors (such as IL-6 and TNF-α) by microglia, and promoting T cell infiltration and neuroinflammation. This mechanism is particularly significant in the context of *APOE*ε4. The TRPV1 activator capsaicin can inhibit the activity of the NF-κB pathway in *APOE*ε4 microglia [126]. Additionally, inflammatory stimuli can trigger the formation of LDs in microglia. In the mouse microglial cell line BV2 model, lipopolysaccharide (LPS) activates the NF-κB pathway, upregulates lipid synthesis-related genes (such as ACLY and PLIN3), and inhibits lipid catabolism genes (such as ADRB1/2), leading to abnormal fatty acid β-oxidation and LDs accumulation [75]. In *APOE*ε4/iPSC-derived microglia, the proportion of lipid droplet-positive cells is significantly higher than that in *APOE*ε3 cells. After interferon-γ (IFN-γ) stimulation, the lipid droplet positivity rate in *APOE*ε4 microglia further increases, with a higher amplitude than that in *APOE*ε3 cells [129]. Research has found that LPS promotes the transcription and translation of LDLR in microglia by activating the TLR4/NF-κB pathway. *APOE*ε4 synergistically enhances this process. Increased LDLR expression enhances cellular cholesteryl ester uptake, ultimately leading to lysosomal dysfunction and the exacerbation of neuroinflammation. This mechanism exhibits a clear genotypic dependency (*APOE*ε4 > ε3 > ε2) [133]. Moreover, studies demonstrate that LPS treatment upregulates ACSL1 expression in iPSC-derived microglia, an effect potentiated by the *APOEε4* genotype. Mechanistically, *APOE*ε4 synergizes with LPS to enhance the transcriptional activation of the *ACSL1* gene by promoting the activation of the NF-κB pathway [76] (Figure 4).

LDs accumulation in microglia also detrimentally affects neurons. On one hand, LDs accumulation in microglia, such as the high LDs burden state induced by *APOE*ε4, significantly inhibits its purinergic signaling pathway (e.g., P2RY12 receptor-mediated Ca^2+^ response), resulting in microglia being unable to effectively sense soluble factors such the as ATP and glutamate released by neurons, thereby losing the ability to dynamically monitor neuronal network activity. On the other hand, *APOE*ε4 exacerbates lipid metabolic disorders in microglia, prompting abnormal cholesterol efflux into the extracellular environment. Excessive cholesterol enhances the activity of G protein-gated inwardly rectifying potassium channels (GIRK3) on the neuronal cell membrane, leading to hyperpolarization of the neuronal resting membrane potential and a decrease in the frequency of action potential firing. Additionally, cholesterol enrichment promotes abnormal aggregation of neuronal lipid rafts, further interfering with synaptic transmission efficiency [105]. Furthermore, microglia with abundant LDs induce tau phosphorylation and apoptosis in neurons by secreting lipid droplet-associated neurotoxic factors (e.g., pro-inflammatory cytokines, lipid metabolites), and this process is dependent on the presence of APOE [76].

## 3. The Role of APOEε2 in Microglia Function Associated with AD

The existing literature shows that, compared to *APOE*ε3 and *APOE*ε4 microglia, *APOE*ε2 microglia demonstrate stronger protective functions in the pathological environment of AD, particularly in terms of maintaining brain homeostasis, regulating neuroinflammation, and effectively clearing Aβ deposits. *APOE*ε2 may regulate microglial functional activities (including motility, phagocytosis, and immunomodulation) through modulating the expression of microglia-associated proteins. Specifically, *APOE*ε2 is significantly associated with high expression of ionized calcium-binding adapter molecule 1 (Iba1, a marker of microglial motility) and macrophage scavenger receptor-A (MSR-A, a plaque-associated phagocytic receptor), while it is significantly associated with low expression of cluster of differentiation 68 (CD68, a marker of phagocytic function) and human leukocyte antigen–DR (HLA-DR, an antigen-presenting molecule). These results suggest that *APOE*ε2 may exert neuroprotective effects by preserving microglial motility (associated with Iba1 expression) and alleviating neuronal damage induced by elevated CD68 and HLA-DR expression [51]. Moreover, compared to *APOE*ε4 microglia, *APOE*ε2 microglia exhibit lower activation levels upon inflammatory stimulation, secrete fewer pro-inflammatory cytokines, and induce less neuronal damage [87]. Following LPS stimulation, *APOE*ε2 microglia exhibit attenuated activation of the p38 MAPK signaling pathway [52]. Furthermore, in microglial predominant gene clusters rich in biological processes such as engulfment, inflammatory response, cell–cell interaction, and metabolism, the expression levels of specific genes within this cluster are lower in *APOE*ε2 carriers than in *APOE*ε3 homozygotes, regardless of the presence of neuritic amyloid plaques [89]. Similarly, APOE2 stimulation reduces the expression levels of microglial activation markers TREM2 and Clec7a, suggesting its ability to suppress the transition of microglia into disease-associated activation states (e.g., DAM/MGnD phenotype) [54]. These findings demonstrate that *APOE*ε2 plays a critical role in maintaining microglial immune surveillance function while sustaining a relatively low inflammatory activation state, thereby reducing potential neurotoxic damage caused by excessive inflammatory activation.

Compared to APOE3 or APOE4, APOE2 significantly enhances the microglial degradation of soluble Aβ. This enhanced degradative capacity may be attributed to the structural and functional properties of APOE2, which potentially accelerates Aβ clearance by interacting with Aβ itself or related degradative enzymes, thereby reducing Aβ accumulation in the brain [55]. A study on macrophages supports this hypothesis. Bone marrow-derived macrophages from APOE2-TR mice demonstrated a significantly enhanced degradation capacity for both soluble and insoluble Aβ species compared to those from APOE3-TR and APOE4-TR mice. Mechanistically, this superior degradative performance may be attributed to APOE2’s ability to potentiate matrix metalloproteinase-9 (MMP-9) enzymatic activity, thereby facilitating more efficient Aβ proteolysis [134]. However, studies examining microglial phagocytic function toward apoptotic neuronal cells and Aβ have shown no significant differences between *APOE*ε2 microglia and wild-type or *APOE*ε3 microglia in terms of their ability to engulf apoptotic neurons or Aβ [61]. This may be due to differences in experimental models and detection methods. Notably, studies have demonstrated that the APOE can directly interact with Aβ by binding to residues 12–28 of the Aβ peptide, an interaction that promotes both Aβ aggregation and cerebral deposition. In both APP/*APOE*ε4 and APP/*APOE*ε2 transgenic mouse models, the co-deposition of APOE with Aβ plaques has been consistently observed. Compared to APOE4, APOE2 does not provide complete protection against Aβ pathology, but rather exerts a more moderate promotive effect on Aβ-related pathological processes [135].

Several lines of evidence indirectly support the role of *APOE*ε2 in modulating microglial function to ameliorate AD pathology. *APOE*ε2 gene therapy significantly reduces the activation of *APOE*ε4 microglia, accompanied by a decrease in Aβ deposition and improvements in neuroinflammation and neurodegenerative changes [27]. These findings provide new insights into the potential clinical applications of *APOE*ε2 gene therapy as a treatment option for AD. In the CPZ-induced demyelination model, *APOE*ε2 microglia demonstrated a robust capacity to clear myelin debris, effectively responding to demyelination injury and thereby facilitating tissue repair [53]. Additionally, researchers have identified genes that are preferentially expressed in aged human microglia, which are significantly enriched in AD susceptibility genes and increase with age. However, *APOE*ε2 reduces the expression of these genes, suggesting that it may help decrease the incidence of the aged microglial phenotype [136].

## 4. The Role of APOEε3 in Microglia Function Associated with AD

*APOE*ε3 microglia demonstrate a unique equilibrium between homeostatic maintenance and pathological response in AD. Using APOE-knockout mice with microglia/central nervous system-associated macrophages (CAMs)-specific inducible expression of *APOE*ε3, researchers discovered that *APOE*ε3 microglia can efficiently perform immunosurveillance in the brain and directionally migrate toward injury sites to form a potential barrier preventing further damage. This rapid-response mechanism is crucial for neuroprotection—it not only contains the spread of injury-induced excessive inflammatory responses but also minimizes damage to surrounding healthy neurons, thereby maintaining homeostasis of the brain microenvironment [46]. Compared to nAPOE4_1–151_, the genes upregulated in microglia following stimulation with nAPOE3_1–151_ (the amino-terminal fragment of APOE3) are associated with various cellular processes, including mitosis, cytoskeleton regulation, and cell signaling. These gene functions are more closely related to the physiological regulation of microglia, supporting their normal functions in maintaining brain homeostasis [98].

*APOE*ε3 expression in microglia has significant potential to improve amyloid deposition and related pathological conditions, increasing the microglial coverage of amyloid plaques and the expression of TREM2 [46,49,107]. The intracellular signaling response triggered by TREM2 activation is considered essential for the effective phagocytosis of Aβ by microglia [109]. In the presence of Aβ, *APOE*ε3 regulates the expression of genes related to antigen presentation, immune response, inflammatory pathways, and interferon response in microglia, leading to a shift toward specific phenotypes (ARM and MGnD microglia). This enhances their positive response to Aβ, increases their phagocytic activity, and modulates the inflammatory response [46,49,56]. During the progression of neurodegenerative lesions, *APOE*ε3 microglia upregulate key DAM/MGnD genes while downregulating TGFβ signaling molecules, thereby reducing the levels of hyperphosphorylated tau protein and ultimately decreasing neuronal loss [56]. Notably, there are notable sex differences in the interactions of *APOE*ε3 microglia with amyloid plaques; compared with age-matched female E3 FAD mice, male E3 FAD mice exhibit greater microglial plaque coverage, plaque compaction, and TREM2 expression, along with a reduced plaque burden [107]. Furthermore, DAM/MGnD microglia expressing *APOE*ε3 can secrete specific factors, such as lectin galactoside-binding soluble 3 (LGALS3), to activate astrocytes, promoting their recruitment to Aβ plaques and the encapsulation of the plaques, thereby limiting the diffusion and toxicity of Aβ [56].

In recent years, researchers have discovered a negative correlation between certain mutations of *APOE*ε3 and the risk of AD. The R136S mutation in *APOE*ε3, known as the *APOE*3 Christchurch variant (*APOE*3ch) or *APOE*3 R136S, enhances the ability of microglia to handle plaque-associated NFTs and tau seeds, thereby reducing tau accumulation in synapses. Additionally, it modulates the uptake of Aβ and tau by microglia, suppresses their inflammatory response, and decreases the toxicity of the exosomes they secrete, providing protective effects against AD [34,137,138,139]. Another extremely rare *APOE*ε3 missense mutation, the *APOE*ε3-Jacksonville variant (*APOE*ε3-Jac, also known as *APOE* p.V236E), can significantly reduce amyloid plaque burden and plaque-associated neurotoxicity, thereby substantially lowering the risk of AD [33,140]. The discovery of these protective *APOE*ε3 variants and elucidation of their neuroprotective mechanisms have identified novel molecular targets and therapeutic approaches for developing AD treatment strategies targeting the APOE–microglial pathway.

## 5. The APOE–Microglia Axis: A Novel Concept in Alzheimer’s Disease Pathogenesis and Therapy

The body of evidence presented in the previous section highlights that *APOE* alleles influence microglial phenotypes, inflammatory responses, phagocytic function, and lipid metabolism, forming a closely interconnected signaling axis—the APOE–microglia axis. This axis reveals complex interactions among genetics, immunity, and metabolism in AD and links the genetic risk of *APOE* alleles to the dynamic functions of microglia. At its core, the APOE–microglia axis involves bidirectional regulation between APOE proteins and microglia: microglia upregulate *APOE* expression in the AD pathological environment, while APOE proteins in turn shape microglial functional states, thereby influencing key AD pathological processes such as amyloid pathology, tau pathology, and neuroinflammation. The three major *APOE* alleles drive functional divergence in microglia by differentially regulating their phenotypes, states, and molecular pathways. Elucidating these mechanisms would both uncover the cellular basis of AD genetic susceptibility and identify critical targets for *APOE* genotype-based personalized therapies.

## 6. Clinical Therapeutic Prospects

The APOE–microglia axis represents a pivotal target for AD therapeutics. Promising clinical applications include *APOE*ε2 gene therapy, which has shown potential in ameliorating AD pathology [27]. Similarly, gene-editing techniques aimed at modifying the *APOE* genotype in microglia could restore normal phagocytic function, enhancing Aβ plaques clearance while mitigating excessive neuroinflammatory responses [29,141]. These approaches aim to reduce APOE4 levels and increase APOE2 levels, which have been demonstrated to exhibit therapeutic efficacy [142]. For *APOE*ε4 carriers, reducing APOE levels has emerged as a viable therapeutic strategy. However, precise control of this reduction is critical to avoid off-target effects [143]. Furthermore, therapeutic strategies targeting APOE-regulated specific sites hold significant promise (Table 3). However, given that AD pathogenesis involves a multifaceted interplay of genetic, epigenetic, and environmental factors, the APOE–microglia axis represents only one component of this intricate network. Thus, targeting it alone is unlikely to fully halt AD progression, underscoring the need for integrated, comprehensive therapeutic strategies.

However, APOE–microglia axis-related therapies also face challenges. It should be noted that *APOE* is expressed throughout the body. Therefore, when applying APOE-targeted drugs in vivo, the greater challenge may lie in off-target effects, necessitating the prioritization of tissue and cell specificity in drug design [147]. Regarding reducing the systemic effects of APOE-targeted drugs, there are multiple research directions worthy of in-depth exploration in the future. From the perspective of drug delivery systems, the development of brain-targeted delivery vectors represents one of the key directions. For instance, by leveraging specific transporters on the blood–brain barrier (BBB), such as transferrin receptors and insulin receptors, drugs targeting APOE can be conjugated to ligands that bind to these transporters. This enables receptor-mediated transcytosis across the BBB, facilitating the preferential enrichment of drugs in the brain and reducing their distribution in other peripheral tissues [148,149]. In terms of gene therapy, viral vectors can be used to achieve brain-specific gene editing or regulation. Adeno-associated virus (AAV) has good safety and low immunogenicity, and there are multiple serotypes, some of which have a high tropism for brain tissues. For example, by modifying the AAV vector to carry therapeutic genes for APOE (such as genes regulating *APOE* expression or correcting the abnormal structure of APOE4) and using brain-specific promoters in promoter selection, the therapeutic genes can only function in the brain, avoiding unnecessary interference with the function of APOE in other tissues throughout the body [27,150].

## 7. Future Research Directions

While the APOE–microglia interplay offers crucial insights into AD mechanisms and therapeutic opportunities, key aspects of this regulatory axis remain elusive. Emerging evidence indicates that microglia harbor multiple signaling pathways that are either regulated by APOE alleles or involved in APOE interactions. Under pathological AD conditions, APOE may dysregulate these pathways, triggering microglial dysfunction that ultimately drives AD progression [56,65,76,80,81,91]. Therefore, systematically elucidating the precise molecular mechanisms of APOE-mediated pathway regulation, identifying novel APOE-regulated pathways, and deciphering their inter-pathway crosstalk are critical for establishing a comprehensive APOE–microglial functional regulatory network in AD. Notably, distinct APOE alleles (ε2/ε3/ε4) may exert differential regulatory effects on these pathways [56,76,91]. In-depth investigation of these allele-specific regulatory mechanisms will provide crucial theoretical insights into AD pathogenesis.

Another crucial area is to dissect the heterogeneous effects of *APOE* alleles on microglial function. Microglial cells exhibit remarkable heterogeneity, enabling them to differentially respond to diverse stimuli (e.g., infection, injury, or degeneration) and thereby enhance the adaptive capacity of the CNS [151]. Current research utilizing single-cell multi-omics technologies can precisely identify distinct phenotypic and functional states of microglia regulated by *APOE* alleles, such as DAM/MGnD microglia [64], LDAM [75], and TIM [73]. The identification of APOE-regulated microglial phenotypes not only provides critical therapeutic targets for developing precision interventions against specific microglial states, but also offers novel biomarkers for monitoring disease progression and evaluating prognosis [64,73,74,75].

Furthermore, microglia do not drive AD pathogenesis in isolation—rather, multiple CNS cell types, including astrocytes and neurons, collectively contribute to disease progression through extensive cross-talk with microglia [152,153]. It is therefore imperative to investigate how *APOE*ε4 modulates these intercellular interactions, rather than focusing solely on its cell-autonomous effects on microglial function [82,154]. Similarly, the inflammatory response, lipid metabolism, and phagocytic functions of microglia are not isolated processes, but rather interconnected and integrated through intricate intracellular signaling pathways and molecular mechanisms. These functional interactions collectively form a comprehensive regulatory network that governs microglia’s physiological and pathological roles in the central nervous system [155]. As previously discussed, APOE exerts holistic regulation over microglial functions. APOE (particularly the APOE4 isoform)-mediated lipid metabolic dysregulation is significantly associated with aberrant inflammatory responses and phagocytic dysfunction, forming an interconnected pathological network. However, the precise molecular mechanisms underlying this multidimensional regulation remain to be elucidated.

Further research into the neuroprotective mechanisms of *APOE*ε2 and *APOE*ε3 is also critical. Current studies demonstrate a pronounced “*APOE*ε4-centric” bias, with severely inadequate exploration of *APOE*ε2 and *APOE*ε3. This imbalance significantly hinders our ability to develop a systematic understanding of the *APOE* family’s comprehensive mechanistic role in AD. It should be noted that *APOE*ε4 represents the ancestral allele conserved in non-human primates and all examined mammalian species. The more pertinent question is why and how *APOE*ε2 and *APOE*ε3 alleles evolved to modify microglial (and other cellular) functions, rather than why *APOE*ε4 exhibits detrimental effects [21]. Moreover, exploring how *APOE*ε2 and *APOE*ε3 modulate microglial activity and their functional differences compared to *APOE*ε4 may reveal new therapeutic targets. Additionally, studying rare *APOE*ε3 variants, such as *APOE*3ch and *APOE*3-Jac, may provide insights into protective mechanisms against AD [33,34,137].

## 8. Conclusions

In the pathogenesis of AD, the APOE–microglia axis serves as a central regulatory hub. Different *APOE* alleles (*APOE*ε2, *APOE*ε3, and *APOE*ε4) profoundly shape AD pathological progression by governing microglial phenotypes, inflammatory signaling, phagocytic capacity, and lipid metabolic homeostasis. Specifically, *APOE*ε4 drives AD progression through a cascade of dysfunctions, including aberrant microglial hyperactivation, impaired Aβ clearance, exacerbated neuroinflammation, and disrupted lipid homeostasis. In contrast, *APOE*ε2 confers neuroprotection by suppressing excessive microglial activation, enhancing Aβ phagocytosis, and attenuating inflammatory responses, thereby reducing AD susceptibility. *APOE*ε3 maintains basal microglial lipid metabolism and inflammatory balance, while rare protective variants (e.g., APOE3ch and APOE3-Jac) further mitigate AD risk by fine-tuning microglial functional profiles. Future investigations should prioritize dissecting the precise molecular mechanisms of the APOE–microglia axis, with a focus on the regulatory roles of *APOE*ε2 and *APOE*ε3. Additionally, elucidating the intercellular crosstalk between microglia and other CNS) cell types mediated by this axis is essential. Such mechanistic insights will provide a robust theoretical foundation for developing genotype-specific therapeutic strategies targeting the APOE-microglia axis in AD.

## Figures and Tables

**Table 1:** Comparative effects of different *APOE* alleles on microglial function.

Microglia Function	*APOE*ε2	*APOE*ε3	*APOE*ε4
Activation states	Mild activation	Homeostatic	Hyperactive
Inflammation	Suppressed	Neutral	Elevated
Aβ clearance	Efficient	Moderate	Impaired
Tau pathology	Protective	Mild	deteriorating
Lipid metabolism	Balanced	Balanced	Disrupted
Migration ability	Normal	Normal	Impaired

**Table 2:** Distinct microglial subpopulations modulated by *APOE*ε4.

Type	Promoting Factors	Inhibiting Factors	Related Genes	Functional Features	Refs.
DAM (MGnD)	APOE-TREM2 axis, Aβ plaques, apoptotic neurons	——	*TREM2*, *APOE*, *TYROBP*, *LPL*	Increased phagocytosis	[64,65]
TIMs	Neuroinflammation, aging, *APOE*ε4, cellular stress	——	*NF-κB*, *C/EBP*, *AP-1*	Impaired phagocytosis/ inflammation	[73]
ARM	Aβ plaques, tau pathology	*APOE*ε4 TREM2-R47H	*CD163*	Increased phagocytosis	[74]
MHC-II microglia	Aβ plaques, tau pathology, Neuronal APOE4	——	*MHC-II*	Increased inflammation/ phagocytosis	[47]
LDAM	Inflammation, *APOE*ε4, Aβ plaques	——	*ACSL1*	Increased inflammation, impaired phagocytosis	[75,76]

Abbreviations: Aβ: amyloid-β; ACSL1: Acyl-CoA synthetase long-chain family member 1; *AP-1*: activator protein 1; ARM: amyloid-responsive microglia; C/EBP: CCAAT/enhancer-binding protein; *CD163*: cluster of differentiation 163; DAM: disease-associated microglia; LDAM: lipid-droplet accumulating microglia; LPL: lipoprotein lipase; *MHC-II*: major histocompatibility complex class II; MGnD: neurodegenerative microglia; *NF-κB*: nuclear factor kappa-light-chain-enhancer of activated B cells; TIMs: terminally inflammatory microglia; *TREM2*: triggering receptor expressed on myeloid cells 2; *TYROBP*: TYRO protein tyrosine kinase binding protein.

**Table 3:** Potential AD therapeutic strategies targeting APOE-microglia axis.

Axis	Therapeutic Target	Intervention	Mechanisms	Refs.
LXR-APOE axis	LXR	LXR agonists (GW3965)	Activation of LXR promotes APOE upregulation, enhancing cholesterol transport and metabolism, and preventing intracellular cholesterol accumulation.	[144,145]
APOE—HIF-1α axis	HIF-1α	HIF-1α inhibitors	*APOE*ε4 upregulates HIF-1α, driving microglia toward a phenotype resembling DAM/MGnD with pro-inflammatory and glycolytic metabolic shifts.	[77,146]
VCAM1-APOE axis	VCAM1	VCAM1 agonists	Microglial VCAM1 expression promoting migration toward APOE-containing Aβ plaques. VCAM1-APOE interaction induces DAM/MGnD transformation, enhancing Aβ clearance.	[83]
APOE4—ITGB8-TGFβ axis	TGFβ	TGFβ inhibitor	The microglial APOE4-ITGB8-TGFβ pathway serves as a negative regulator of microglial response to AD pathology, and restoring the MGnD phenotype via blocking ITGB8-TGFβ signaling provides a promising therapeutic intervention for AD.	[56,65]
TREM2-APOE axis	TREM2, SYK, miR155	TREM2/SYK agonists, miR-15 inhibitor	TREM2-SYK signaling axis activation is essential for the DAM/MGnD phenotype. miR-155 suppresses the downstream IFNγ signaling pathway, thereby blocking the DAM/MGnD response.	[65,80,81]
IL-17F—IL-17RA axis	IL-17F	IL-17F inhibitor	*APOE*ε4-associated neutrophils exhibit elevated IL-17F expression, which engages microglial IL-17RA to inhibit the DAM/MGnD phenotype. Disrupting this IL-17F/IL-17RA axis improved cognitive function in a mouse model of AD.	[82]
APOE—NF-κB/NLRP3 axis	NF-κB, NLRP3	NF-κB/NLRP3 inhibitor,	APOE4 can significantly induce the activation of NF-κB and more effectively activate the NLRP3 inflammasome, enhancing the neuroinflammatory response of microglia.	[91]
APOE—NF-κB ACSL1 axis	ACSL1	ACSL1 inhibitor (Triacin C)	*APOE*ε4 promotes microglial lipid droplet accumulation (LDAM phenotype) through NF-κB-mediated transcriptional activation of ACSL1.	[76]
APOE4- LilrB3 axis	LilrB3	LilrB3 antagonist	The specific interaction between APOE4 protein and LilrB3(an immune checkpoint receptor protein expressed on the surface of microglia) activates microglia, driving their transition into a pro-inflammatory state.	[101]

Abbreviations: Aβ: amyloid-β; ACSL1: Acyl-CoA synthetase long-chain family member 1; AD: Alzheimer’s disease, APOE: apolipoprotein E, DAM: disease-associated microglia; HIF-1α: hypoxia-inducible factor 1-alpha; IFNγ: interferon gamma; IL-17F: interleukin 17F; IL-17RA: interleukin 17 receptor A; ITGB8: integrin subunit beta 8; LDAM: lipid-droplet accumulating microglia; LilrB3: leukocyte immunoglobulin-like receptor B3; LPL: lipoprotein lipase; LXR: liver X receptor; miR-155: microRNA-155; miR-15: microRNA-15; MGnD: neurodegenerative microglia; NF-κB: nuclear factor kappa-light-chain-enhancer of activated B cells; NLRP3: NLR family pyrin domain containing 3; SYK: spleen-associated tyrosine kinase; TGFβ: transforming growth factor beta; TIMs: terminally inflammatory microglia; TREM2: triggering receptor expressed on myeloid cells 2; VCAM1: vascular cell adhesion molecule 1.
